# Prevalence and clinical correlates of focal choroidal excavation in a large cohort of Chinese patients with choroidal osteoma

**DOI:** 10.1186/s40662-025-00452-2

**Published:** 2025-09-01

**Authors:** Yi Xuan, Wenyi Tang, Xiaofeng Ye, Wei Liu, Gezhi Xu, Min Wang, Qing Chang

**Affiliations:** 1 Eye Institute and Department of Ophthalmology, Eye & ENT Hospital, Fudan University, Shanghai, 20031 China; 2 Shanghai Key Laboratory of Visual Impairment and Restoration, Shanghai, 200031 China; 3 NHC Key Laboratory of Myopia and Related Eye Diseases; Key Laboratory of Myopia and Related Eye Diseases, Chinese Academy of Medical Sciences, Shanghai, 200031 China

**Keywords:** Choroidal osteoma, Focal choroidal excavation, Classification, Prevalence

## Abstract

**Purpose:**

To describe the prevalence and clinical characteristics of focal choroidal excavation (FCE) in a large cohort of Chinese patients with choroidal osteoma (CO).

**Methods:**

One hundred and thirty-two eyes of 110 Chinese patients diagnosed with CO were enrolled. The prevalence and clinical characteristics of FCE were studied. Univariate and multivariate linear regression analyses were used to identify the factors associated with the occurrence of FCE. Furthermore, FCEs were divided into two types based on their location: Type 1 (at the edge of the tumor) and Type 2 (inside the tumor), and their clinical features were analyzed.

**Results:**

The prevalence of FCE was 46.2% in 132 eyes with CO. Eyes with FCEs demonstrated a longer disease duration (*P* < 0.01), worse BCVA (*P* = 0.01), longer greatest tumor linear dimension (*P* < 0.01), larger total tumor area (*P* < 0.01) and decalcification area (*P* < 0.01), and a higher incidence of outer retinal tubulation (ORT) (*P* = 0.01). Only disease duration (*P* = 0.025) was significantly correlated with the occurrence of FCE. Patients with Type 2 FCEs had a larger greatest linear dimension of FCEs and a higher likelihood of ORT, choroidal neovascularization, disruption of the external limiting membrane, and inner retina compared with those with Type 1 FCEs (all *P* < 0.05).

**Conclusions:**

The duration is associated with the development of FCE in CO. The different types of FCE may indicate varying stages of CO, suggesting the occurrence and enlargement of FCE in CO are associated with the lateral expansive growth of the tumor. Comprehensive optical coherence tomography evaluation of tumor margins and extramacular regions during initial assessment and regular follow-up is recommended to enable early FCE detection (particularly Type 2), allowing timely identification of CNV and other complications for prompt vision-preserving intervention.

**Supplementary Information:**

The online version contains supplementary material available at 10.1186/s40662-025-00452-2.

## Background

Focal choroidal excavation (FCE) was first described by Jampol et al. based on optical coherence tomography (OCT) findings [1]. It is characterized by a depression of the choroid and external retina, without accompanying scleral ectasia or posterior staphyloma. FCE can be congenital/primary, mostly observed in myopic individuals, and typically presents without evidence of other chorioretinal diseases. Alternatively, it can be acquired/secondary, associated with various vision-threatening chorioretinal disorders, including pachychoroid diseases, chorioretinal inflammation, dystrophies, and choroidal tumors such as choroidal osteoma (CO) [2].

A recent study indicated that CO was the most common etiology for FCE [3], which typically affects young females. Gass and colleagues in 1978 described this as a unique, mostly unilateral intraocular tumor composed of mature bone within the peripapillary or macular choroid [4]. Despite its benign nature, CO can lead to significant vision-threatening problems, primarily due to tumor enlargement, decalcification, atrophy of the overlying retina, and secondary choroidal neovascularization (CNV) [5]. Several cases of CO have been reported in association with FCE. Olguin-Manríquez et al. [6] reported that 2 out of 17 CO eyes (12.5%) developed FCE, while Seong et al. [7] found FCE present in 23 out of 41 CO eyes (52.3%). However, these two studies did not study FCE features in detail. The prevalence and clinical characteristics of FCE in Chinese patients with CO remain undetermined, and no prior studies have investigated potential factors associated with FCE development in CO. The rarity of the disease has made it challenging to draw definitive conclusions regarding the role of FCE in CO.

Therefore, our study aimed to investigate the prevalence rate of FCE in a large Chinese cohort with CO and to identify the factors associated with FCE in these patients, enhancing our understanding of the pathophysiology underlying the formation of FCE in CO.

## Methods

The cross-sectional study was approved by the Institutional Review Board of the Eye and ENT Hospital of Fudan University (Reference No. 2020052), and all procedures were conducted in accordance with the tenets of the Declaration of Helsinki. Informed consent was obtained from all participants or their legal guardians.

One hundred and thirty-two eyes of 110 patients diagnosed with CO were recruited from July 2014 to May 2024 at a tertiary referral center in East China (Eye & ENT Hospital of Fudan University, Shanghai, China). All patients underwent comprehensive clinical and imaging assessments, including best-corrected visual acuity (BCVA), slit-lamp fundus ophthalmoscopy, fundus photography (Topcon TRC50LX; Topcon, Tokyo, Japan; CLARUS 500; Carol Zeiss, Dublin, CA; Optos 200Tx, Dunfermline, Scotland, United Kingdom), B-scan ultrasonography, spectral-domain OCT (SD-OCT) using enhanced depth imaging (EDI) mode, fundus autofluorescence, fluorescein angiography (FFA) and indocyanine green angiography (ICGA) simultaneously by Spectralis HRA + OCT (Heidelberg Engineering, Heidelberg, Germany), and OCT angiography (OCTA) (AngioVue, RTVue-XR Avanti, Optovue Inc., Fremont, CA, USA; VG200S, SVision Imaging, Henan, China).

**Fig. 1:**
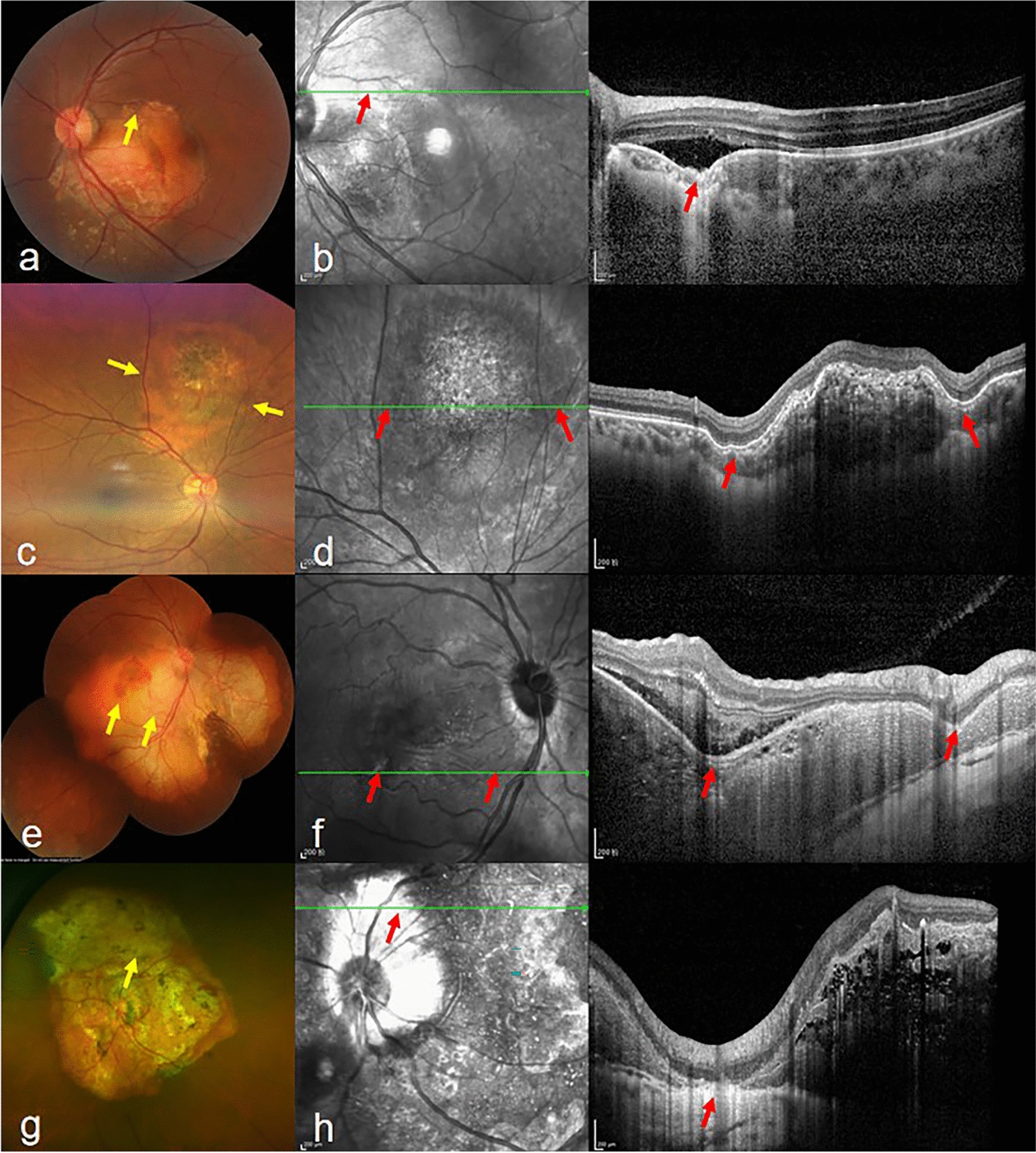
Classification of focal choroidal excavation (FCE) in choroidal osteoma (CO). **a**-**d** Type 1. This type is located at the edge of CO. **a**, **b** A 28-year-old male with nonconforming FCE, visible as a focal depression (yellow arrow, color fundus photograph, **a**) with corresponding optical coherence tomography (OCT) findings (red arrows, en face and B-scan images, **b**). **c**, **d** A 60-year-old female with conforming FCE, demonstrating choroidal excavation (yellow arrows, color fundus photograph, **c**) and intact overlying retinal layers (red arrows, OCT en face and B-scan images, **d**). **e**–**h** Type 2. This type is situated inside the tumor and can be one or multiple focal areas of choroidal depression, with retinal pigment epithelium (RPE) and outer retinal irregularity. **e**, **f** A 39-year-old female with dual FCEs (one nonconforming, one conforming), highlighted on mosaic fundus photography (yellow arrows, **e**) and OCT (red arrows, en face and B-scan images, **f**). **g**, **h** A 36-year-old female with conforming FCE (yellow arrow, ultrawide-field fundus photograph, **g**) showing RPE, outer retinal, and inner retinal irregularity on OCT (red arrows, **h**)

The demographic data included patient age at diagnosis, sex, BCVA at the initial visit, laterality (unilateral or bilateral), patient-reported presenting symptoms and disease duration (weeks) between the onset of symptoms and presentation to the hospital, ocular history, and previous treatment. Tumor characteristics included tumor location (macula, peripapillary, or peripapillary with macular involvement), the greatest tumor linear dimension, the greatest height, and the total and decalcified areas of each tumor. The decalcification evaluated by fundus photography, OCT and autofluorescence was defined as an atrophic yellow-white region with overlying RPE and choriocapillaris atrophy, corresponding to areas of hyper-autofluorescence or hypo-autofluorescence, depending on the degree of degeneration as previously reported [8–10]. The areas were calculated manually by outlining the tumor boundary using the Image J software (https://imagej.nih.gov/ij/) by two independent retinal specialists (YX and WYT). The results were obtained by analyzing the mean values of two measurements, after which liability was also estimated. The overlying retinal features anywhere within the range of the tumor were recorded which included the presence of CNV, intraretinal fluid (IRF), subretinal fluid (SRF), intraretinal hemorrhage (IRH), outer retinal tubulation (ORT), hyperreflective retinal dots (HRDs) and pitchfork sign which was characteristic of hyperreflective vertical projections into the outer retina mimicking pitchfork tines [11]. OCTA was utilized in conjunction with OCT to ascertain the presence of CNV, and the fluorescent leakage of CNV on FFA and ICGA indicated active lesions. Data regarding the areas above the FCE were also noted, including the presence of CNV or ORT, and the condition of the retinal pigment epithelium (RPE), ellipsoid zone (EZ), interdigitation zone (IZ), and external limiting membrane (ELM). FCE was detected using Spectralis HRA + OCT. Each FCE was assessed by EDI-OCT, using raster sections through the fovea and tumor. We defined FCE as a specific downward deflection of the Bruch’s membrane-RPE complex line on OCT. FCEs were classified as conforming or nonconforming type using standardized OCT criteria [12]. Conforming FCE was defined as the absence of any detectable separation between the photoreceptor tips and the RPE on OCT. In contrast, nonconforming FCE was characterized by distinct detachment of photoreceptors from the RPE, evidenced by a hyporeflective space intervening between these layers on OCT. The greatest linear dimension (GLD) and depth of each FCE were manually measured with a built-in caliper tool in the Heidelberg OCT device software. All measurements were taken by two retinal specialists (YX and WYT), and the average of the two measurements was recorded for further analysis. We classified FCE into two types based on its location relative to the tumor (Fig. 1): Type 1 (at the edge of the tumor), and Type 2 (inside the tumor). For Type 1, FCE was located around the elevated tumor, and we chose the maximum value of GLD and depth of FCE. For Type 2, FCE could be one or multiple focal areas of choroidal depression inside the tumor, and we also chose the maximum values of GLD and depth of FCE if there were multiple FCEs. Two masked retinal specialists (YX and WYT) independently classified FCE as Type 1 or Type 2 based on SD-OCT and fundus photography. Interobserver agreement was also assessed using the Kappa (κ) with 95% confidence intervals (CI). Levels of agreement were evaluated: 0–0.20, poor; 0.21–0.40, fair; 0.41–0.60, moderate; 0.61–0.80, substantial; and 0.81–1.00, almost perfect agreement [13]. The results showed that the interobserver agreement for FCE classification was almost perfect (κ = 0.83, 95% CI: 0.69–0.96), supporting the reproducibility of the proposed typology.

Descriptive statistics were used to characterize the study population. The spherical equivalent of refractive error was analyzed. Continuous variables were summarized as mean ± standard deviation and categorical variables with counts and percentages. The visual acuity was converted to the logarithm of the minimal angle of resolution (logMAR) for statistical analyses. Between-group differences were assessed using unpaired *t*-test for continuous variables, and χ^2^ test or Fisher’s exact test for categorical variables as appropriate. Univariate and multivariate linear regression analyses were performed to determine the factors associated with FCE in CO patients. Statistical analysis was performed using SPSS statistical software (version 21, IBM, Armonk, NY). *P* values of less than 0.05 were considered statistically significant.

## Results

### Characteristics of patients with CO

A total of 132 eyes from 110 patients were included in the study. Patient demongraphics and tumor features are summarized in Table 1. Among the patients, there were 39 males and 71 females (M/F ratio, 1:1.82). The mean age at diagnosis was 34.8 ± 14.2 years (range: 4 to 73 years). Fourteen patients (12.7%) were children (< 18 years old), with a mean age of 11.9 ± 4.2 years and a M/F ratio of 4/10. Involvement was noted in the right eye for 67 patients and the left eye for 65 patients, with 22 patients (20%) having bilateral COs. At the first visit, the mean baseline logMAR BCVA was 0.63 ± 0.60 [20/86, range: hand movement (HM) to 20/20]. The most common presenting symptom was decreased vision, reported in 42.4% of eyes, followed by blurred vision (22.7%), metamorphopsia (18.2%), and central scotoma (6.1%). Almost one-tenth of patients (10.6%) reported no visual symptoms. Seventeen eyes had a history of other retinochoroidal diseases, with central serous chorioretinopathy (CSC) accounting for 3.8% and uveitis for 4.5%. Three eyes (2.3%) underwent photodynamic therapy while five eyes (3.8%) received anti-VEGF treatment due to CNV and SRF. In total, there were 139 tumors, with seven eyes (5.3%) displaying two tumors in a single eye. The macular area was affected in over half of the tumors. Tumor decalcification was observed in 95.5% of the eyes with a mean onset duration of 70.2 ± 144.0 weeks (range: 0.25 to 720 weeks). Measurements of the tumor, including its GLD, height, total tumor area, and total decalcification area, are detailed in Table 1.

**Table 1:** Baseline patient characteristics with choroidal osteomas (n = 110 patients) with or without focal choroidal excavation

Parameters	Overall	FCE (+)	FCE (−)	*P*
No. of eyes (%)	132	61 (46.2)	71 (53.8)	NA
Age (years, range)	34.8 ± 14.2 (4–73)	35.6 ± 12.8 (10–60)	34.0 ± 15.3 (4–73)	0.55
No. of female patients (%)	71 (64.5)	34 (68.0)	37 (61.7)	0.49
Laterality (OD, %)	67 (50.8)	33 (54.1)	34 (47.9)	0.48
Refractive error (SE, D)	− 1.1 ± 2.4 (− 14.0 to 7.5)	− 1.5 ± 2.3 (− 7.0 to 2.0)	− 0.8 ± 2.5 (− 14.0 to 7.5)	0.08
Symptom (no. of eyes, %)				
Vision decrease	56 (42.4)	26 (42.6)	30 (42.2)	0.97
Blurring	30 (22.7)	12 (19.7)	18 (25.4)	0.44
Metamorphopsia	24 (18.2)	15 (24.6)	9 (12.7)	0.08
Central scotoma	8 (6.1)	3 (4.9)	5 (7.0)	0.61
None	14 (10.6)	5 (8.2)	9 (12.7)	0.40
Duration (week)	70.2 ± 144.0	115.1 ± 190.3	31.6 ± 66.8	< 0.01
LogMAR BCVA (Snellen)	0.63 ± 0.60 (20/86, HM to 20/20)	0.78 ± 0.65 (20/121, HM to 20/20)	0.50 ± 0.50 (20/64, CF to 20/20)	0.01
With two COs (no. of eyes, %)	7 (5.3)	5 (8.2)	2 (2.8)	0.17
CO location (no. of tumors, %)				
Macular	62 (44.6)	27 (40.9)	35 (47.9)	0.40
Peripapillary	37 (26.6)	15 (22.7)	22 (30.1)	0.32
Peripapillary-macular	40 (28.8)	24 (36.4)	16 (21.9)	0.06
Tumor size				
Linear length (mm)	7.9 ± 4.5	9.6 ± 4.6	6.5 ± 4.0	< 0.01
Height (μm)	989.9 ± 400.0	950.1 ± 416.3	854.8 ± 383.1	0.17
Area (mm^2^)	52.2 ± 68.9	71.4 ± 85.3	33.8 ± 44.5	< 0.01
Decalcification (no. of eyes, %)	126 (95.5)	60 (98.4)	66 (93.0)	0.29
Decalcification area (mm^2^)	28.3 ± 51.7	42.7 ± 66.2	16.0 ± 30.3	< 0.01
CNV (no. of eyes, %)	52 (39.4)	28 (45.9)	24 (33.8)	0.16
Active CNV (no. of CNV, %)	41 (78.8)	21 (75.0)	20 (83.3)	0.46
SRF (no. of eyes, %)	88 (66.7)	41 (67.2)	47 (66.2)	0.90
IRF (no. of eyes, %)	33 (25.0)	20 (32.8)	13 (18.3)	0.06
IRH (no. of eyes, %)	32 (24.2)	19 (59.4)	13 (18.3)	0.08
HRD (no. of eyes, %)	83 (62.8)	38 (62.3)	45 (63.4)	0.90
ORT (no. of eyes, %)	31 (23.5)	21 (34.4)	10 (14.1)	0.01
Ocular history (no. of eyes, %)				
CSC	5 (3.8)	1 (1.6)	4 (5.6)	0.25
AMD	1 (0.8)	0	1 (1.4)	1.00
PCV	1 (0.8)	1 (1.6)	0	0.46
Uveitis	6 (4.5)	1 (1.6)	5 (7.0)	0.14
Optic neuritis	1 (0.8)	0	1 (1.4)	1.00
Glaucoma	1 (0.8)	1 (1.6)	0	0.46
IOIP	1 (0.8)	1 (1.6)	0	0.46
BRVO	1 (0.8)	1 (1.6)	0	0.46
Previous treatment (no. of eyes, %)				
Anti-VEGF treatment	5 (3.8)	2 (3.0)	3 (4.1)	0.79
Photodynamic therapy	3 (2.3)	1 (1.5)	2 (2.7)	0.67
Eyes with 2 FCEs	9 (6.8)	9 (14.8)	NA	NA

*FCE* = focal choroidal excavation; *NA* = not applicable; *SE* = spherical equivalent; *D* = diopter; *BCVA* = best-corrected visual acuity; *CO* = choroidal osteoma; *CNV* = choroidal neovascularization; *SRF* = subretinal fluid; *IRF* = intraretinal fluid; *IRH* = intraretinal hemorrhage; *HRD* = hyperreflective dot; *ORT* = outer retinal tubulation; *CSC* = central serous chorioretinopathy; *AMD* = age-related macular degeneration; *PCV* = polypoidal choroidal vasculopathy; *IOIP* = idiopathic orbital pseudotumor; *BRVO* = branch retinal vein occlusion; *VEGF* = vascular endothelial growth factor

### Correlation between FCE and underlying CO

A total of 70 FCEs were identified among the 139 tumors, present in 61 eyes out of 132 eyes (resulting in a prevalence of 46.2%). Of all the eyes with FCEs, six eyes (9.8%) were identified to have additional complicating ocular diseases. Comparisons between eyes with and without FCEs are shown in Table 1. Eyes with FCEs exhibited a longer disease duration (*P* < 0.01), worse BCVA (*P* = 0.01), longer greatest tumor linear dimension (*P* < 0.01), a larger total tumor area (*P* < 0.01), a larger decalcification area (*P* < 0.01), and a higher incidence of ORT (*P* = 0.01). Further univariate and multivariable regression analyses revealed that only the disease duration (*P* = 0.025) was significantly associated with the occurrence of FCE (Table 2).

**Table 2:** Factors associated with focal choroidal excavation in choroidal osteoma

Independent variable	Univariate			Multivariate		
	Exp (B)	95% CI	*P*	Exp (B)	95% CI	*P*
Age	1.007	0.983 to 1.032	0.58	-	-	-
Gender	1.510	0.735 to 3.145	0.27	-	-	-
Laterality (OD, %)	1.465	0.738 to 2.934	0.28	-	-	-
Refractive error (SE, D)	1.143	0.955 to 1.397	0.16	-	-	-
Disease duration	1.005	1.002 to 1.010	0.01	1.004	1.001 to 1.009	0.04
LogMAR BCVA	2.327	1.259 to 4.572	0.01	1.168	0.532 to 2.621	0.70
CO location	1.409	0.941 to 2.128	0.10	-	-	-
Tumor size						
Linear length (mm)	1.181	1.084 to 1.299	< 0.01	1.136	0.964 to 1.345	0.13
Height (μm)	1.001	0.999 to 1.002	0.17	-	-	-
Area (mm^2^)	1.011	1.004 to 1.020	0.01	0.9981	0.980 to 1.018	0.84
Decalcification area (mm^2^)	1.018	1.006 to 1.035	0.01	1.003	0.982 to 1.028	0.78
CNV	1.662	0.824 to 3.381	0.16	-	-	-
SRF	1.047	0.506 to 2.177	0.90	-	-	-
ORT	3.203	1.395 to 7.775	0.01	1.435	0.488 to 4.253	0.51
HRD	0.955	0.470 to 1.944	0.90	-	-	-
IRF	2.176	0.983 to 4.961	0.06	-	-	-
IRH	2.398	1.070 to 5.569	0.04	2.323	0.927 to 5.974	0.07

*SE* = spherical equivalent; *D* = diopter; *BCVA* = best-corrected visual acuity; *CO* = choroidal osteoma; *CNV* = choroidal neovascularization; *SRF* = subretinal fluid; *ORT* = outer retinal tabulation; *HRD* = hyperreflective dot; *IRF* = intraretinal fluid; *IRH* = intraretinal hemorrhage

### Clinical and morphological features of FCEs in CO

**Fig. 2:**
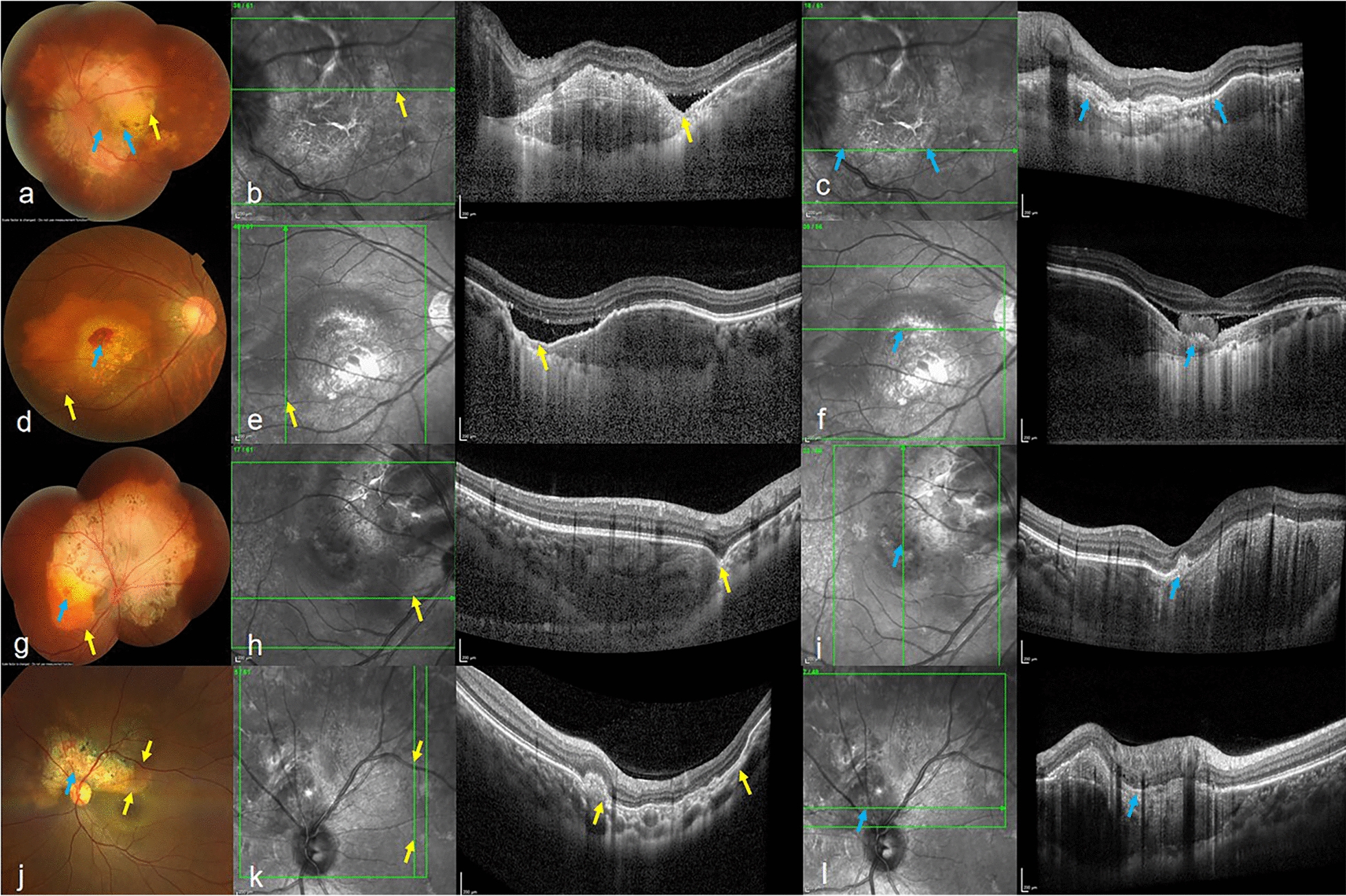
Representative cases demonstrating coexisting Type 1 and Type 2 focal choroidal excavations (FCEs) in choroidal osteoma (CO) patients. **a**-**c** Peripapillary CO in a 56-year-old male showing: (**a**) Mosaic color fundus photograph showing Type 1 (yellow arrow) and Type 2 (blue arrows) FCEs; (**b**) Corresponding optical coherence tomography (OCT) en face and B-scan images of Type 1 FCE; (**c**) OCT en face and B-scan images of Type 2 FCE. **d-f** Macular-involving CO with subretinal hemorrhage in a 46-year-old female: (**d**) Fundus photograph showing both FCE types; (**e**) Type 1 FCE on OCT; (**f**) Type 2 FCE on OCT. **g**-**i** Superior-papillary CO involving macula in a 24-year-old female: (**g**) Mosaic color fundus photograph showing both FCE types; (**h**) Type 1 FCE on OCT; (**i**) Type 2 FCE on OCT. **j**-**l** Superior-papillary CO in a 38-year-old female: (**j**) Fundus photograph showing both FCE types; (**k**) Type 1 FCE on OCT; (**l**) Type 2 FCE on OCT

**Fig. 3:**
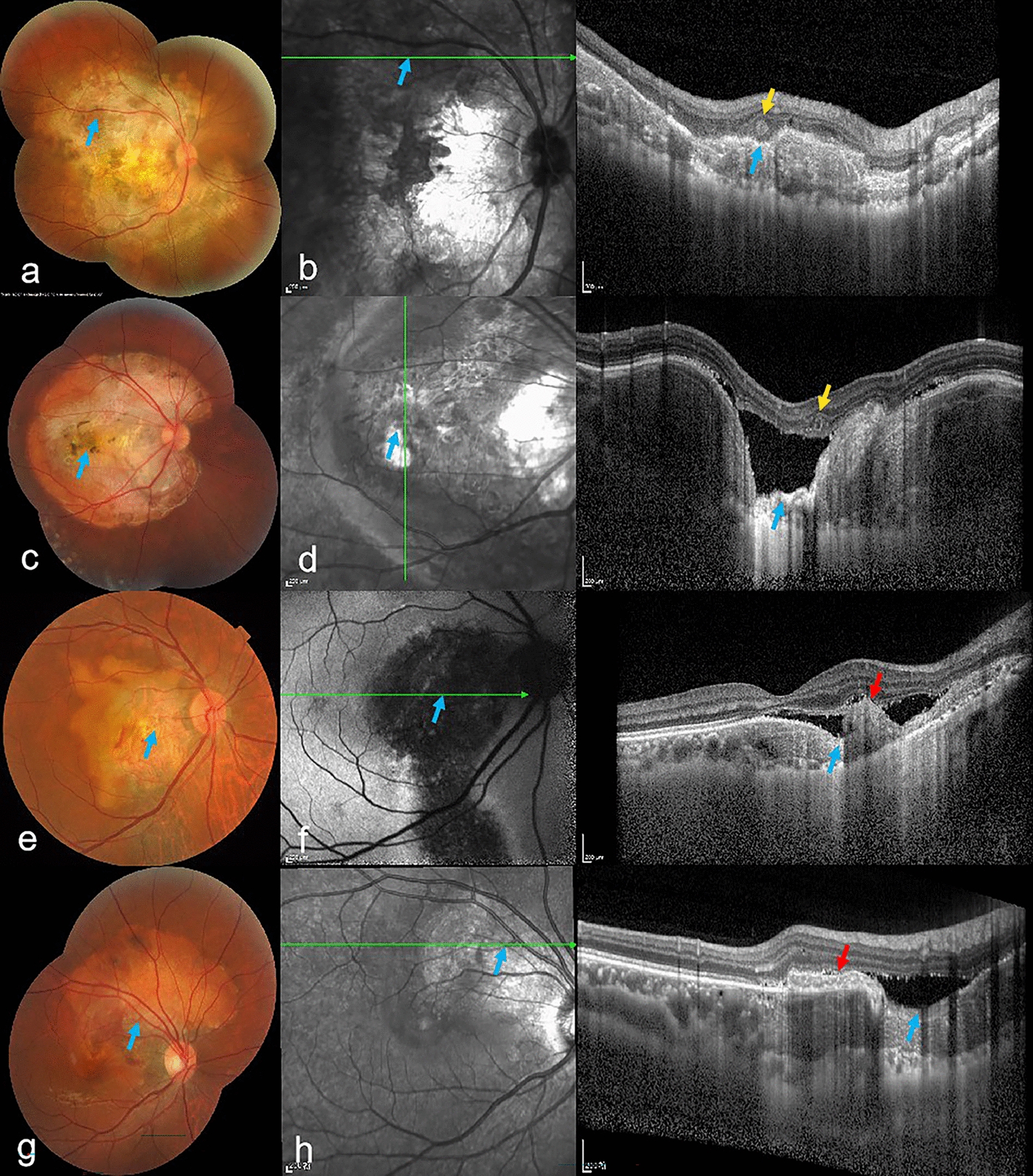
Choroidal osteoma (CO) cases demonstrating focal choroidal excavation (FCE) with associated outer retinal tubulation (ORT) and choroidal neovascularization (CNV). **a**, **b** Peripapillary CO in a 45-year-old female with: (**a**) Mosaic fundus image showing Type 2 FCE (blue arrow); (**b**) Optical coherence tomography (OCT) en face and B-scan images revealing ORT (yellow arrow) within the conforming FCE. **c**, **d** Macular-involving peripapillary CO in a 13-year-old female with: (**c**) Mosaic fundus image showing Type 2 FCE; (**d**) OCT en face and B-scan images demonstrating ORT at nonconforming FCE boundary. **e**, **f** Macular CO in a 54-year-old female with: (**e**) Fundus image showing Type 2 FCE; (**f**) Fundus autofluorescence and B-scan OCT revealing CNV (red arrow) at nonconforming FCE base. **g**, **h** Superior peripapillary CO in a 29-year-old male with: (**g**) Mosaic fundus image showing Type 2 FCE; (**h**) B-scan OCT demonstrating CNV on nonconforming FCE slope

**Fig. 4:**
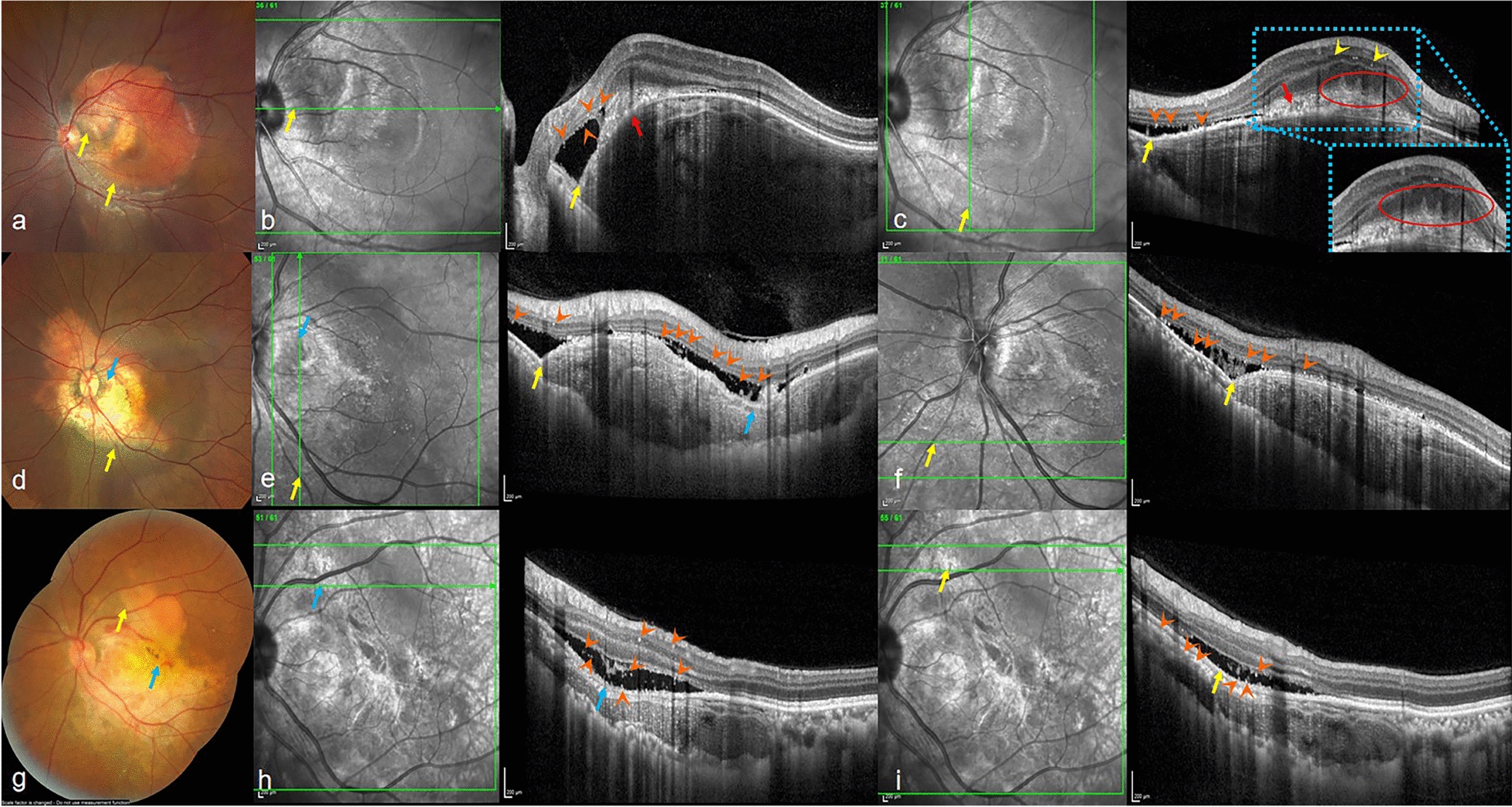
Imaging features of choroidal osteoma (CO) with focal choroidal excavations (FCEs), pitchfork sign, and hyperreflective dots (HRDs). **a–c** Macular CO with Type 1 FCE and associated choroidal neovascularization (CNV) in a 23-year-old female. **a** Color fundus photograph showing a Type 1 FCE (yellow arrows). **b, c** Corresponding optical coherence tomography (OCT) en face and B-scan images demonstrating: neovascular membrane (red arrow, **b**) on the slope of the FCE; the "pitchfork sign" (red circle, **c**) visible on the surface of the neovascularization; and scattered HRDs (orange arrowheads, **b** and **c**) in the inner nuclear layer and at the subretinal fluid/retinal pigment epithelium (RPE) interface. **d**–**f** Peripapillary CO with Type 1 and Type 2 FCEs in a 35-year-old female. **d** Color fundus image highlighting Type 1 FCE (yellow arrow) and Type 2 FCE (blue arrow). **e, f** OCT revealing HRDs (orange arrowheads) at the subretinal fluid border, RPE junction, and inner nuclear layer, localized within both FCE regions. **g–i** Temporal juxtapapillary-macular CO with mixed FCE types in a 45-year-old female. **g** Mosaic fundus image depicting Type 1 FCE (yellow arrow) and Type 2 FCE (blue arrow). **h**, **i** OCT demonstrating HRDs (orange arrowheads) in the ganglion cell layer, inner nuclear layer, and subretinal fluid/RPE interface, spanning both FCE foci

Among all FCEs, 31 were classified as Type 1 and 39 as Type 2. Seven eyes exhibited both Type 1 and Type 2 FCEs (Fig. 2). In 19 eyes with Type 1 and 15 eyes with Type 2, a hyporeflective space was observed between the tips of the photoreceptors and the underlying RPE, indicating a nonconforming FCE; conversely, in the remaining 36 eyes, no apparent separation was noted between the photoreceptor tips and the RPE, implying a conforming FCE. Neovascular complexes were found in 17 eyes, located on the slope or at the bottom of choroidal excavation. ORT was observed within the boundary of FCE in 15 eyes (Fig. 3). HRDs were detected scattered throughout all retinal layers in 38 eyes and were distributed at the border of SRF and cystoid spaces or close to the RPE layer in five eyes. The "pitchfork sign" was noted on the surface of CNV in two eyes (Fig. 4).

We analyzed clinical characteristics, including FCE parameters, the occurrence of CNV, ORT, HRD, SRF, IRF, IRH, and the condition of the inner and outer retina between Type 1 and Type 2 FCEs (Table 3). The results revealed statistically significant differences in the GLD of FCEs, the integrity of ELM and inner retina, the presence of ORT within the boundary of FCE, and CNV on the slope or at the bottom of FCE between the two types of FCE (all *P* < 0.05). Patients with Type 2 FCE exhibited larger GLD of FCEs and had a higher likelihood of ORT, CNV and disruption of ELM and inner retina.

**Table 3:** Clinical and morphological features of different types of focal choroidal excavation in choroidal osteoma

Parameters	FCE	Type 1	Type 2	*P*
No. of FCEs	70	31	39	NA
FCE GLD (μm)	2273.9 ± 1270.3	1749.6 ± 1042.6	2690.7 ± 1292.1	< 0.01
FCE Type (C: UC)	36/34	12/19	24/15	0.06
FCE depth (μm)	274.8 ± 332.4	259.9 ± 464.2	286.7 ± 173.8	0.74
RPE disrupted (n, %)	64 (91.4)	27 (87.1)	37 (94.9)	0.25
EZ disrupted (n, %)	68 (97.1)	30 (96.8)	38 (97.4)	1.00
IZ disrupted (n, %)	68 (97.1)	30 (96.8)	38 (97.4)	1.00
ELM disrupted (n, %)	59 (84.3)	23 (74.2)	36 (92.3)	0.04
Inner retina intact (n, %)	31 (44.3)	21 (67.7)	10 (25.6)	< 0.01
Under or near CNV (n, %)	17 (24.3)	3 (9.7)	14 (35.9)	0.01
ORT above FCE (n, %)	15 (21.4)	1 (3.2)	14 (35.9)	< 0.01
With HRD (n, %)	38 (54.3)	13 (41.9)	22 (56.4)	0.23
With SRF n, (%)	41 (58.6)	19 (61.3)	23 (59.0)	0.84
With IRF (n, %)	20 (28.6)	6 (19.4)	14 (35.9)	0.13
With IRH (n, %)	19 (27.1)	11 (35.5)	8 (20.5)	0.16

*FCE* = focal choroidal excavation; *n* = number of FCEs; *NA* = not applicable; *GLD* = greatest linear dimension; *C* = conforming; *UC* = unconforming; *CO* = choroidal osteoma; *RPE* = retinal pigment epithelium; *EZ* = ellipsoid zone; *IZ* = interdigitation zone; *ELM* = external limiting membrane; *CNV* = choroidal neovascularization; *ORT* = outer retinal tubulation; *HRD* = hyperreflective dot; *SRF* = subretinal fluid; *IRF* = intraretinal fluid; *IRH* = intraretinal hemorrhage

## Discussion

FCE is a unique clinical entity that has gained considerable prevalence with the widespread application of OCT. This phenomenon may indicate an underlying retinochoroidal disease, but it remains relatively poorly understood. Several case series have documented FCE in eyes with CO. However, these studies have typically involved small sample sizes and have been based on relatively simple descriptive analyses [14, 15]. In this study, we investigated the clinical characteristics of FCE associated with CO in a large Chinese cohort and evaluated the relationship between FCE and CO. Our findings revealed a prevalence of 46.2% for FCE in eyes with CO, which falls within the range of the previous reports (12.5% [6] and 52.3% [7]). In contrast, the prevalence of FCE in conditions such as CSC [16, 17], PCV [18], age-related macular degeneration (AMD)-CNV [18, 19], multifocal choroiditis and panuveitis (MCP)/punctate inner choroidopathy (PIC) [20] has been reported to be significantly lower, at 6% to 7.8%, 6%, 1% to 4.9%, and 20%, respectively. Our findings suggest that FCE may be a significant clinical feature in patients with CO. It should be noted, however, that several osteoma cases with FCEs in our series were complicated by concurrent ocular pathologies including CSC, PCV, and uveitis. Of note, imaging analysis demonstrated that in all cases of CO coexisting with CSC or PCV, FCEs were strictly confined within the osteoma boundaries (Supplementary Fig. 1). This anatomical colocalization was observed in 100% of comorbid cases, suggesting that FCE development is intrinsically associated with the osteoma itself rather than being secondary to CSC or PCV pathology. Nevertheless, future longitudinal studies with larger patient cohorts are warranted to establish the temporal relationship between FCE formation and comorbid conditions, and investigate potential modifying effects of these comorbidities on FCE progression.

Importantly, this is the first study to explore the correlations of FCE formation in CO patients. We found that the mean disease duration in CO eyes with FCEs was significantly longer than in those without FCEs (115.1 weeks vs. 31.6 weeks). Furthermore, univariate and multivariate linear regression analysis indicate that only the disease duration is significantly associated with the occurrence of FCE. This is not surprising as CO can lead to complications such as decalcification and CNV, both of which have been reported to play a critical role in the formation of FCEs during the progression of the tumor. As one of the most common complications of CO, CNV occurs in approximately 31% to 47% of cases within 10 years [5, 21] and has been shown to frequently accompany FCE, either as a consequence or as a contributing factor to its development [22, 23]. In this study, OCTA was used to detect CNV in CO patients. It turned out that OCTA showed unique advantages in visualizing neovascular networks despite tumor-related structural distortion, thereby facilitating the detection of secondary CNV. CNV foci have been observed within or adjacent to FCEs over time, consistent with our findings. Additionally, decalcification of the tumor is cited as a common pathogenic pathway for the development of FCE and CNV in CO. Several case reports have indicated that most FCEs coincide with the decalcification of CO [3, 6]. The progression of CO begins with tumor growth and calcification, eventually expanding over time to decalcification in the later stages of the disease. As the disease advances and the tumor develops, the choroidal tissue is replaced by mature cancellous bone, which undergoes spontaneous decalcification, resulting in the structural attenuation of both the choroid and retina [4, 24]. The weakening of the RPE-Bruch’s membrane complex and the inner choroid make them susceptible to deformation under exerted stress. Moreover, the mechanical disturbances resulting from fibrosis related to CNV or CO decalcification impose a strong retraction force on the overlying retina, pulling retinal tissues backward during the FCE process [3, 23, 25]. Our findings support the hypothesis that FCE is a distinct and common clinical sign that occurs and evolves as CO progresses.

**Fig. 5:**
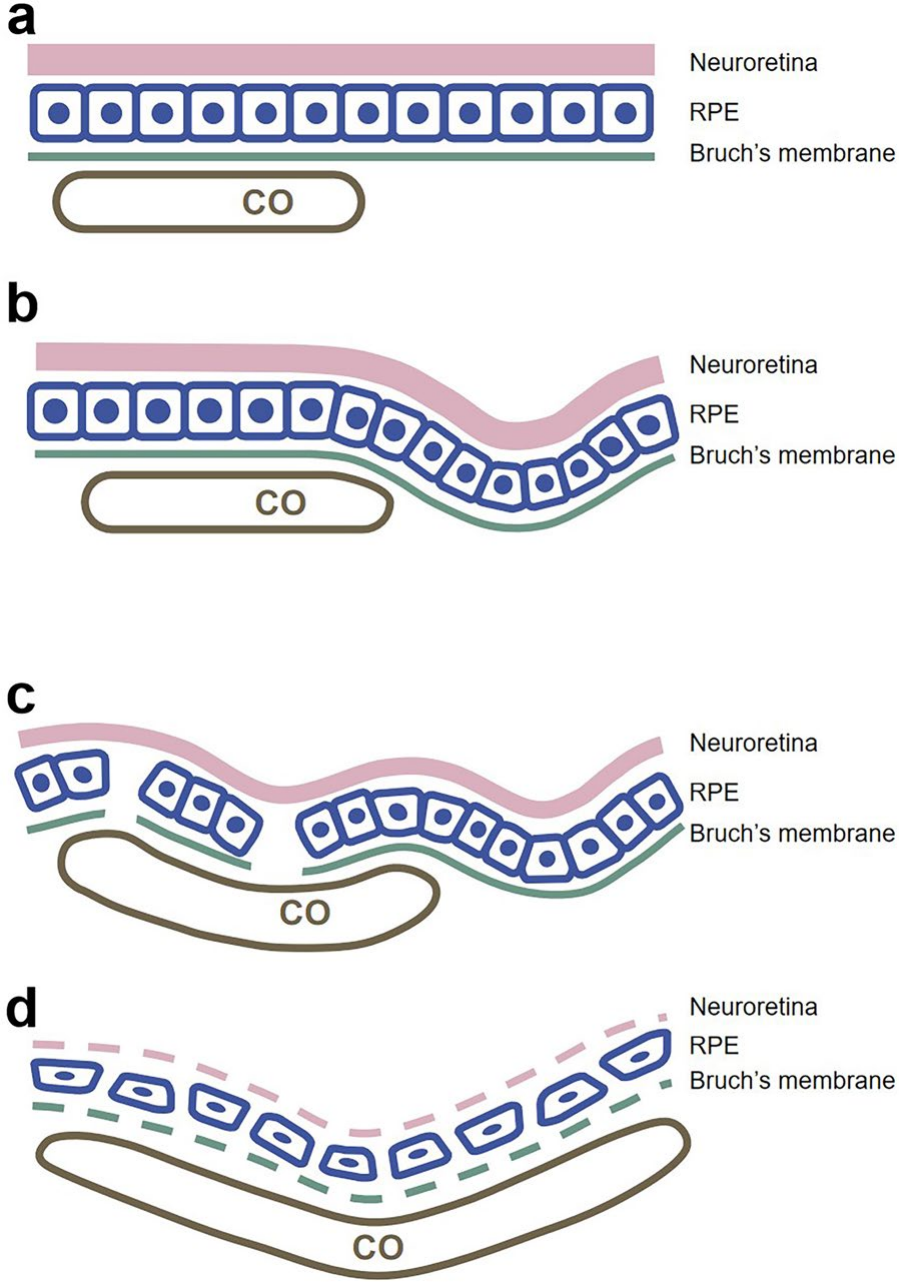
Proposed mechanism for the development and progression of focal choroidal excavation (FCE) in choroidal osteoma (CO). **a** A CO with intact overlying Bruch’s membrane, retinal pigment epithelium (RPE), and neuroretinal layers. **b** As the CO grows and expands, an FCE gradually forms at the CO border, with the overlying Bruch’s membrane, RPE, and neuroretinal layers remaining intact (Type 1 FCE). **c** With further CO expansion, the overlying Bruch’s membrane, RPE and neuroretinal layer undergo progressive thinning and disruption, leading to the formation of a new FCE within the CO (Type 2 FCE). **d** Over time, the Type 2 FCE deepens and enlarges, resulting in atrophy of the overlying Bruch’s membrane, RPE, and neuroretinal layers. Eventually, it can merge with the pre-existing Type 1 FCE at the CO border, forming a larger, confluent FCE

We subdivided FCE patterns in CO into two types (Type 1 and Type 2) based on their location relative to the tumor, which may correspond to different stages of tumor evolution. The most commonly used classification categorizes FCE as conforming and nonconforming types [12]. Shinojima et al. [26] have described three patterns namely, cone-shaped, bowl-shaped, and mixed types, based on their morphology as visualized by OCT in conditions such as AMD, CSC, PCV, and CNV. Obata et al. [27] classified FCEs as foveal or extrafoveal based on whether or not the center of the fovea is involved in cavitation. FCEs with foveal involvement and nonconforming morphology tend to have a higher incidence of visual symptoms. However, these current classifications do not adequately reveal the causative etiology and potential progression of FCE in patients with CO. Our results highlighted the differences in the GLD of FCE, the integrity of ELM and inner retinal layers, as well as the presence of ORT and CNV between FCEs located at the margin of the tumor and those situated inside the tumor. As the natural progression of CO, the gradual decalcification of CO is known to cause progressive defects in both outer and inner retinal tissues and to trigger the formation of CNV and ORT structures [28, 29]. Consequently, this classification of FCE in CO may reflect different stages of tumor development. It can be speculated that the emergence and enlargement of FCE in CO are linked to the dimensional growth and expansion of the tumor. Initially, FCE would be found at the border of an osteoma (Type 1). As the tumor expands, it progressively damages the overlying RPE-Bruch membrane complex and the outer retina, eventually affecting the inner retina leading to the formation of a new FCE inside the tumor (Type 2). This newly formed FCE will deepen and enlarge over time, ultimately merging with the pre-existing FCE at the tumor’s edge, converting into a larger FCE (Fig. 5). Based on our findings, we recommend modifying current imaging protocols to include systematic OCT evaluation of both tumor margins and extramacular intratumoral regions, particularly in peripapillary CO cases. This expanded scanning approach enables earlier detection of Type 1 FCEs at tumor margins while improving identification of Type 2 FCEs within the tumor. Given the demonstrated associations between Type 2 FCEs and complications including ORT, CNV, and structural damage to the ELM and inner retina, we emphasize the necessity of regular follow-up examinations to facilitate early CNV detection and prompt therapeutic intervention.

In addition, HRDs were found to be scattered throughout all retinal layers, often located at the boundary of SRF and cystoid spaces, or near the RPE layer in eyes with Type 1 and Type 2 FCEs in our study. These HRDs appear as round or oval lesions with a discrete, clear boundary and a signal intensity equal to or greater than RPE, measuring 20 to 40 μm in diameter. They have been reported in cases of diabetic retinopathy and retinal vein occlusion and are currently considered to represent activated microglia in an inflammatory environment [30, 31]. Hence, the presence of HRDs indicates active inflammation of the retina. Moreover, this study observed that two cases of FCE were accompanied by a “pitchfork sign” structure on the surface, which is believed to be unique to inflammatory CNV, and similar findings have been reported in cases of CO [32, 33]. These two OCT biomarkers appeared in both Type 1 and Type 2 FCEs, suggesting that the inflammatory response may also be an important driver for the overall development of FCE in CO. Furthermore, these inflammatory biomarkers (e.g., HRDs, pitchfork sign) may provide potential avenues for differentiating inflammatory *vs.* degenerative pathophysiology in FCE formation.

Several limitations of this study should be acknowledged. First, this is a cross-sectional study. While this classification system provides a structural framework for characterizing FCE subtypes, its prognostic implications for long-term visual outcomes or CNV risk require validation through longitudinal studies with serial follow-up. Further, this typology requires prospective validation before being used for clinical decision-making. A subset of our cohort is still in follow-up, and ongoing prospective longitudinal studies will help determine whether FCE can serve as a reliable predictor of disease progression–findings that will be crucial for developing evidence-based monitoring protocols. Second, we exclusively enrolled Chinese participants (the largest CO series to date), which may limit generalizability to other ethnic groups. It has been reported that the average subfoveal choroidal thickness was thinner in Malay participants compared with Chinese and Indian groups, respectively [34]. The macular retina and central choroid of Ghanaians are also significantly thinner as compared with those of European ancestry [35]. These anatomical differences may influence FCE characteristics and warrant validation in diverse populations. Third, although we meticulously collected medical histories, accurate determination of symptom onset remains particularly challenging in pediatric cases. To address this, we performed univariate and multivariable regression analyses after excluding patients under 18 years of age. Notably, only disease duration was significantly associated with FCE occurrence (Supplementary Table 1), suggesting that pediatric disease duration estimates were sufficiently reliable and did not materially affect our primary conclusions. Fourth, it is critical to explicitly contextualize the generalizability of our findings. As a study conducted exclusively in a tertiary referral center, our reported FCE prevalence of 46.2% is not representative of community or screening populations. The high prevalence observed here likely reflects the selective referral of complex cases to specialized centers, thereby limiting direct extrapolation of these results to broader populations. Future multi-tier studies (primary-to-tertiary) are needed to establish epidemiologic profiles. Finally, while SD-OCT cube scans provided adequate FCE visualization, swept source-OCT with 3D reconstruction could offer superior topographic details. Future longitudinal studies employing 3D OCT protocols may provide more detailed insights into FCE progression dynamics and associated risks, such as CNV development.

## Conclusion

We found a significant prevalence of 46.2% of FCE in a large cohort of Chinese patients with CO. The disease duration was significantly associated with the presence of FCE. We classified two types of FCE based on their location relative to the tumor, which may indicate varying stages of CO. Thus, the emergence and enlargement of FCE in CO are thought to be associated with horizontal growth and expansion of the tumor. Comprehensive OCT evaluation of tumor margins and extramacular regions during initial assessment and regular follow-up is recommended to enable early FCE detection (particularly Type 2), allowing timely identification of CNV and other complications for prompt vision-preserving intervention.

## Supplementary Information

Additional file1: Figure S1. Representative images of a 46-year-old female patient with choroidal osteoma (CO) and concurrent polypoidal choroidal vasculopathy (PCV). **a** Fundus photography revealed that the two CO lesions (blue arrows) were located superonasal and superotemporal to the optic disc. **b** Simultaneous indocyanine green angiography (ICGA)-optical coherence tomography (OCT) imaging identified the characteristic polypoidal lesion (yellow arrows) along with its accompanying branching vascular network within the foveal area. **c** Spectral-domain OCT (SD-OCT) revealed that the focal choroidal excavation (FCE) lesion, indicated by the red arrows, was localized within the boundaries of the osteoma, distant from the PCV lesion. Table S1. Factors associated with focal choroidal excavation in choroidal osteoma in this study except patients (< 18 years old)

## Data Availability

The data that support the findings of this study are available from the corresponding author upon reasonable request.

## Abbreviations

FCE: Focal choroidal excavation
OCT: Optical coherence tomography
OCTA: Optical coherence tomography angiography
CO: Choroidal osteoma
CNV: Choroidal neovascularization
IRF: Intraretinal fluid
SRF: Subretinal fluid
IRH: Intraretinal hemorrhage
ORT: Outer retinal tubulation
HRDs: Hyperreflective retinal dots
RPE: Retinal pigment epithelium
EZ: Ellipsoid zone
IZ: Interdigitation zone
ELM: External limiting membrane
GLD: Greatest linear dimension

## References

[1] Jampol LM, Shankle J, Schroeder R, Tornambe P, Spaide RF, Hee MR. Diagnostic and therapeutic challenges. Retina. 2006;26(9):1072–6. 17151497 10.1097/01.iae.0000248819.86737.a5

[2] Verma S, Kumar V, Azad S, Bhayana AA, Surve A, Kumar S, et al. Focal choroidal excavation: review of literature. Br J Ophthalmol. 2021;105(8):1043–8. 32788327 10.1136/bjophthalmol-2020-316992

[3] Gan Y, Ji Y, Zuo C, Su Y, Liao N, Zhang X, et al. Correlation between focal choroidal excavation and underlying retinochoroidal disease: a pathological hypothesis from clinical observation. Retina. 2022;42(2):348–56. 34608106 10.1097/IAE.0000000000003307 PMC8765213

[4] Gass JD, Guerry RK, Jack RL, Harris G. Choroidal osteoma. Arch Ophthalmol. 1978;96(3):428–35. 629679 10.1001/archopht.1978.03910050204002

[5] Shields CL, Sun H, Demirci H, Shields JA. Factors predictive of tumor growth, tumor decalcification, choroidal neovascularization, and visual outcome in 74 eyes with choroidal osteoma. Arch Ophthalmol. 2005;123(12):1658–66. 16344436 10.1001/archopht.123.12.1658

[6] Olguin-Manríquez F, Enríquez AB, Crim N, Meraz-Gutierrez M, Soberón-Ventura V, Ávila I, et al. Multimodal imaging in choroidal osteoma. Int J Retina Vitreous. 2018;4:30. 30128167 10.1186/s40942-018-0132-0 PMC6092861

[7] Seong HJ, Kim YJ, Choi EY, Lee J, Byeon SH, Kim SS, et al. Complications, treatments, and visual prognosis of choroidal osteomas. Graefes Arch Clin Exp Ophthalmol. 2022;260(5):1713–21. 34762167 10.1007/s00417-021-05487-4

[8] Sisk RA, Riemann CD, Petersen MR, Foster RE, Miller DM, Murray TG, et al. Fundus autofluorescence findings of choroidal osteoma. Retina. 2013;33(1):97–104. 22718153 10.1097/IAE.0b013e31825c1cde

[9] Alameddine RM, Mansour AM, Kahtani E. Review of choroidal osteomas. Middle East Afr J Ophthalmol. 2014;21(3):244–50. 25100910 10.4103/0974-9233.134686 PMC4123278

[10] Navajas EV, Costa RA, Calucci D, Hammoudi DS, Simpson ER, Altomare F. Multimodal fundus imaging in choroidal osteoma. Am J Ophthalmol. 2012;153(5):890–5.e3. 22265155 10.1016/j.ajo.2011.10.025

[11] Hoang QV, Cunningham ET Jr, Sorenson JA, Freund KB. The “pitchfork sign” a distinctive optical coherence tomography finding in inflammatory choroidal neovascularization. Retina. 2013;33(5):1049–55. 23514797 10.1097/IAE.0b013e31827e25b8

[12] Margolis R, Mukkamala SK, Jampol LM, Spaide RF, Ober MD, Sorenson JA, et al. The expanded spectrum of focal choroidal excavation. Arch Ophthalmol. 2011;129(10):1320–5. 21670327 10.1001/archophthalmol.2011.148

[13] Landis JR, Koch GG. The measurement of observer agreement for categorical data. Biometrics. 1977;33(1):159–74. 843571

[14] Chawla R, Azad SV, Takkar B, Sharma A, Kashyap B. Nonconforming deep focal choroidal excavation in a patient with choroidal osteoma: a diagnostic dilemma. Ophthalmic Surg Lasers Imaging Retina. 2017;48(11):944–7. 29121366 10.3928/23258160-20171030-12

[15] Introini U, Casalino G, Parodi MB, Bandello F, London NJ. Diagnostic and therapeutic challenges. Retina. 2016;36(2):422–7. 26066702 10.1097/IAE.0000000000000591

[16] Ellabban AA, Tsujikawa A, Ooto S, Yamashiro K, Oishi A, Nakata I, et al. Focal choroidal excavation in eyes with central serous chorioretinopathy. Am J Ophthalmol. 2013;156(4):673–83. 23831223 10.1016/j.ajo.2013.05.010

[17] Luk FO, Fok AC, Lee A, Liu AT, Lai TY. Focal choroidal excavation in patients with central serous chorioretinopathy. Eye. 2015;29(4):453–9. 25853402 10.1038/eye.2015.31 PMC4816359

[18] Lim FP, Wong CW, Loh BK, Chan CM, Yeo I, Lee SY, et al. Prevalence and clinical correlates of focal choroidal excavation in eyes with age-related macular degeneration, polypoidal choroidal vasculopathy and central serous chorioretinopathy. Br J Ophthalmol. 2016;100(7):918–23. 26504178 10.1136/bjophthalmol-2015-307055

[19] Kuroda Y, Tsujikawa A, Ooto S, Yamashiro K, Oishi A, Nakanishi H, et al. Association of focal choroidal excavation with age-related macular degeneration. Invest Ophthalmol Vis Sci. 2014;55(9):6046–54. 25190653 10.1167/iovs.14-14723

[20] Kim H, Woo SJ, Kim YK, Lee SC, Lee CS. Focal choroidal excavation in multifocal choroiditis and punctate inner choroidopathy. Ophthalmology. 2015;122(7):1534–5. 25687028 10.1016/j.ophtha.2015.01.012

[21] Aylward GW, Chang TS, Pautler SE, Gass JD. A long-term follow-up of choroidal osteoma. Arch Ophthalmol. 1998;116(10):1337–41. 9790633 10.1001/archopht.116.10.1337

[22] Azimizadeh M, Hosseini SM, Babaei E. Focal choroidal excavation in a case of choroidal osteoma associated with choroidal neovascularization. J Ophthalmic Vis Res. 2020;15(3):419–23. 32864073 10.18502/jovr.v15i3.7461 PMC7431724

[23] Basavaraj TM, Galiyugavaradhan S. Sequential imaging of a case of choroidal osteoma using swept-source OCT and optical coherence tomography angiography: a 4-year follow-up study. Indian J Ophthalmol. 2019;67(12):2097–100. 31755474 10.4103/ijo.IJO_919_19 PMC6896548

[24] Xuan Y, Wang M, Chang Q, Zhang Y. Swept-source optical coherence tomography analysis of choroidal osteoma. Chin J Ocul Fundus Dis. 2020;36(6):435–41.

[25] Olguin-Manríquez F, Enríquez AB, Crim N, Meraz-Gutierrez M, Soberón-Ventura V, Ávila I, et al. Multimodal imaging in choroidal osteoma. Int J Retina Vitreous. 2018;4:30. 30128167 10.1186/s40942-018-0132-0 PMC6092861

[26] Shinojima A, Kawamura A, Mori R, Yuzawa M. Morphologic features of focal choroidal excavation on spectral domain optical coherence tomography with simultaneous angiography. Retina. 2014;34(7):1407–14. 24830823 10.1097/IAE.0000000000000108

[27] Obata R, Takahashi H, Ueta T, Yuda K, Kure K, Yanagi Y. Tomographic and angiographic characteristics of eyes with macular focal choroidal excavation. Retina. 2013;33(6):1201–10. 23514801 10.1097/IAE.0b013e31827b6452

[28] Xuan Y, Zhang Y, Wang M, Guo J, Li L, Liu W, et al. Multimodal fundus imaging of outer retinal tubulations in choroidal osteoma patients. Retina. 2018;38(1):49–59. 28098734 10.1097/IAE.0000000000001498

[29] Xuan Y, Chang Q, Zhang YJ, Ye XF, Liu W, Li L, et al. Clinical observation of choroidal osteoma using swept-source optical coherence tomography and optical coherence tomography angiography. Appl Sci. 2022;12(9):4472.

[30] Bolz M, Schmidt-Erfurth U, Deak G, Mylonas G, Kriechbaum K, Scholda C. Optical coherence tomographic hyperreflective foci: a morphologic sign of lipid extravasation in diabetic macular edema. Ophthalmology. 2009;116(5):914–20. 19410950 10.1016/j.ophtha.2008.12.039

[31] Ebneter A, Kokona D, Schneider N, Zinkernagel MS. Microglia activation and recruitment of circulating macrophages during ischemic experimental branch retinal vein occlusion. Invest Ophthalmol Vis Sci. 2017;58(2):944–53. 28170538 10.1167/iovs.16-20474

[32] Rajabian F, Arrigo A, Grazioli A, Sperti A, Bandello F, Battaglia Parodi M. Focal choroidal excavation and pitchfork sign in choroidal neovascularisation associated with choroidal osteoma. Eur J Ophthalmol. 2021;31(2):NP67–70. 31813301 10.1177/1120672119892802

[33] de Mello PC, Berensztejn P, Brasil OF. Re: The “pitchfork sign” a distinctive optical coherence tomography finding in inflammatory choroidal neovascularization. Retina. 2015;35(3):e23–4. 25699507 10.1097/IAE.0000000000000565 PMC4975298

[34] Song Y, Tham YC, Chong C, Ong R, Fenner BJ, Cheong KX, et al. Patterns and determinants of choroidal thickness in a multiethnic Asian population: The Singapore Epidemiology of Eye Diseases Study. Ophthalmol Retina. 2021;5(5):458–67. 32858246 10.1016/j.oret.2020.08.012

[35] Zouache MA, Faust CD, Silvestri V, Akafo S, Lartey S, Mehta R, et al. Retinal and choroidal thickness in an indigenous population from Ghana: comparison with individuals with European or African ancestry. Ophthalmol Sci. 2024;4(2):100386. 37868802 10.1016/j.xops.2023.100386 PMC10585639

