# Supplementary material for: Prevalence and clinical correlates of focal choroidal excavation in a large cohort of Chinese patients with choroidal osteoma
Source: Eye Vis (Lond). 2025 Sep 1;12:35. doi: 10.1186/s40662-025-00452-2 (PMC12400656; doi:10.1186/s40662-025-00452-2)
Supplement: Supplementary file 1 — Additional file1: Figure S1. Representative images of a 46-year-old female patient with choroidal osteoma (CO) and concurrent polypoidal choroidal vasculopathy (PCV). a Fundus photography revealed that the two CO lesions (blue arrows) were located superonasal and superotemporal to the optic disc. b Simultaneous indocyanine green angiography (ICGA)-optical coherence tomography (OCT) imaging identified the characteristic polypoidal lesion (yellow arrows) along with its accompanying branching vascular network within the foveal area. c Spectral-domain OCT (SD-OCT) revealed that the focal choroidal excavation (FCE) lesion, indicated by the red arrows, was localized within the boundaries of the osteoma, distant from the PCV lesion. Table S1. Factors associated with focal choroidal excavation in choroidal osteoma in this study except patients (< 18 years old) [file 40662_2025_452_MOESM1_ESM.docx]

**Supplementary Figure S1.** Representative images of a 46-year-old female patient with choroidal osteoma (CO) and concurrent polypoidal choroidal vasculopathy (PCV). **a** Fundus photography revealed that the two CO lesions (blue arrows) were located superonasal and superotemporal to the optic disc. **b** Simultaneous indocyanine green angiography (ICGA)- optical coherence tomography (OCT) imaging identified the characteristic polypoidal lesion (yellow arrows) along with its accompanying branching vascular network within the foveal area. **c** Spectral-domain OCT (SD-OCT) revealed that the FCE lesion, indicated by the red arrows, was localized within the boundaries of the osteoma, distant from the PCV lesion.

**
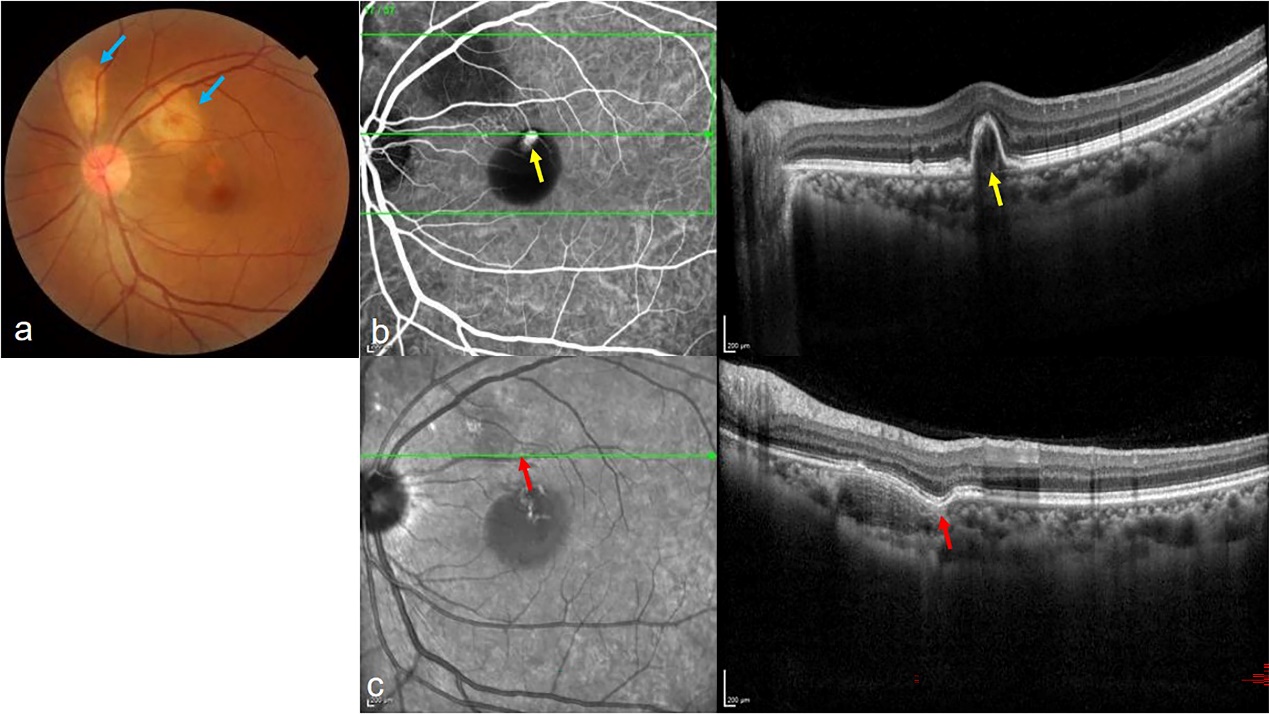
**

**Supplementary Table S1.** Factors associated with focal choroidal excavation in choroidal osteoma in this study except patients (< 18 years old)

|  | **Univariate** | | |  | **Multivariate** | | |
| --- | --- | --- | --- | --- | --- | --- | --- |
| **Independent Variable** | **Exp(B)** | **95% CI** | ***P*** |  | **Exp(B)** | **95% CI** | ***P*** |
| Age | 0.996 | 0.965 to 1.028 | 0.81 |  | - | - | - |
| Gender | 1.451 | 0.673 to 3.128 | 0.34 |  | - | - | - |
| Laterality (OD, %) | 1.476 | 0.707 to 3.081 | 0.30 |  | - | - | - |
| Refractive error (SE, D) | 1.137 | 0.925 to 1.399 | 0.22 |  | - | - | - |
| Disease duration | 1.006 | 1.002 to 1.010 | < 0.05 |  | 1.004 | 1.001 to 1.009 | <0.05 |
| LogMAR BCVA | 2.882 | 1.393 to 5.960 | < 0.01 |  | 1.368 | 0.548 to 3.418 | 0.50 |
| CO location | 1.395 | 0.910 to 2.139 | 0.13 |  | - | - | - |
| Tumor size |  |  |  |  |  |  |  |
| Linear length (mm) | 1.227 | 1.107 to 1.359 | < 0.01 |  | 1.136 | 0.964 to 1.345 | 0.07 |
| Height (μm) | 1.001 | 1.000 to 1.002 | 0.08 |  | - | - | - |
| Area (mm^2^) | 1.013 | 1.004 to 1.022 | 0.01 |  | 0.998 | 0.977 to 1.019 | 0.83 |
| Decalcification area (mm^2^) | 1.021 | 1.004 to 1.038 | 0.02 |  | 1.002 | 0.977 to 1.028 | 0.87 |
| CNV | 1.791 | 0.847 to 3.787 | 0.13 |  | - | - | - |
| SRF | 0.812 | 0.371 to 1.778 | 0.60 |  | - | - | - |
| ORT | 3.431 | 1.355 to 8.686 | 0.01 |  | 1.220 | 0.371 to 4.014 | 0.74 |
| HRD | 0.875 | 0.406 to 1.886 | 0.73 |  | - | - | - |
| IRF | 2.351 | 0.997 to 5.546 | 0.05 |  | - | - | - |
| IRH | 2.751 | 1.175 to 6.441 | 0.02 |  | 2.183 | 0.804 to 5.927 | 0.13 |

SE = spherical equivalent; D = diopter; BCVA = best-corrected visual acuity; CO = choroidal osteoma; CNV = choroidal neovascularization; SRF = subretinal fluid; ORT = outer retinal tubulation; HRD = hyperreflective dot; IRF = intraretinal fluid; IRH = intraretinal hemorrhage
